# Vitamin D and curcumin-loaded PCL nanofibrous for engineering osteogenesis and immunomodulatory scaffold

**DOI:** 10.3389/fbioe.2022.975431

**Published:** 2022-08-08

**Authors:** Abdullrahman M. Al-Bishari, Bilal A. Al-Shaaobi, Aisha A. Al-Bishari, Mohammed A. Al-Baadani, Liang Yu, Jiating Shen, Lei Cai, Yiding Shen, Zhennan Deng, Peng Gao

**Affiliations:** ^1^ School and Hospital of Stomatology, Wenzhou Medical University, Wenzhou, China; ^2^ College of Dentistry, University of Science & Technology, Sanaa, Yemen; ^3^ School Hospital of Stomatology, Zhejiang Chinese Medical University, Hangzhou, China

**Keywords:** electrospinning, vitamin D, curcumin, anti-inflammatory, bone regeneration

## Abstract

The accelerating bone healing process is still a major challenge in clinical orthopedics, especially in critical-sized bone defects. Recently, Nanofiber membranes are showing increasing attention in the biomedical field due to their good biocompatibility, mechanical stability, and the ability to work as a drug carrier to achieve localized and sustained drug delivery. Herein, a multifunction nanofiber membrane loaded with vitamin D (Vit D) and curcumin (Cur) was successfully fabricated using electrospinning technology. In addition, we innovatively modified Vit D with PEG to improve the hydrophilicity of PCL nanofibers. The vitro results of CCK-8, alkaline phosphatase (ALP) and mineralization demonstrated that the PCL/Vit D-Cur membrane had great potential for enhancing the proliferation/differentiation of osteoblasts. Moreover, the synergistic effect of Vit D-Cur loaded PCL nanofiber membrane showed a superior ability to improve the anti-inflammatory activity through M2 polarization. Furthermore, *in vivo* results confirmed that the defect treated with PCL/Vit D-Cur nanofiber membrane was filled with the newly formed bone after 1 month. These results indicate that the Vit D/Cur loaded membrane can be applied for potential bone regeneration therapy.

## 1 Introduction

Various materials have been used for bone replacements to repair human skeleton defects and abnormalities (Critchley et al., 2020). To effectively repair bone defects and abnormalities, scaffolds play an important role in bone support and regeneration by acting as templates for host cells (Bisht et al., 2021). A good scaffold must be biodegradable, biocompatible, and have similar functions to extracellular matrix (ECM) (Asghari et al., 2017). Recently, electrospinning has been utilized to fabricate fibrous scaffolds for biomedical purposes since these electrospinning scaffolds allow the creation of three-dimensional structures similar to that of natural ECM (Rahmati et al., 2021). The size and structure of electrospinning scaffolds can be controlled by adjusting the characteristics of the solution and operating conditions. Cell adhesion, migration, and proliferation can be greatly enhanced due to the high surface area to volume of electrospinning scaffolds (Zanutto et al., 2019; Shokrani et al., 2022). In addition to the biomaterials used, electrospinning techniques are also used in various materials, including synthetic and natural polymers (Puertas-Bartolomé et al., 2021). Many of them have been used in different biomedical applications, including poly (lactic-co-glycolic acid) (PLGA), poly lactic acid (PLA), polycaprolactone (PCL), dextran, aniline (AN), chitosan, chitin, silk fibroin (SF), alginate, and fibrinogen (Nilforoushzadeh et al., 2020; Nourbakhsh et al., 2020; Rahmati et al., 2021). A common synthetic hydrophobic biodegradable polymer that takes a long time to break down in the human body is known as polycarprolactone (PCL) (He et al., 2017; Heydari et al., 2017). Several studies have shown that the slow degradation rate makes it useful for a time-controlled release of medications over an extended period of time (Castro et al., 2018; Liu et al., 2020; Vonbrunn et al., 2020; Al-Baadani et al., 2021). Therefore, it has been used mainly as a long-term implant material for drug release (especially hydrophilic drugs) and supports bone formation (Al-Bishari et al., 2021). It has come to our understanding that vitamin D (Vit D) plays a major role in maintaining bone mineral density in the human body. The inadequacy of Vit D levels in the body can increase the risk of bone-related problems, such as fractures and osteoporosis (Lips and Van Schoor, 2011). In the elderly, osteoporosis and its related fractures are becoming more prevalent, associated with decreased levels of Vit D (Feldman et al., 2013). Furthermore, according to previous studies, Vit D correlates with periodontal tissue health. For instance, the risk of bone-related periodontal diseases decreases when an optimum level of Vit D is present. The role of this naturally synthesized essential nutrient is to modulate bone mineralization for bone density maintenance. Therefore, regulating Vit D levels using external applications is vital to prevent and mitigate bone-related diseases (Feldman et al., 2013; de Sire et al., 2022; Levkovitz et al., 2022).

In complimentary to Vit D, curcumin (Cur, bis-1,7-[4-hydroxy-3-]-hepta-1,6-dione) is a natural, synthetic yellow polyphenol compound extracted from the genus Curcuma (Gunes et al., 2016). Cur is also well known for its anti-inflammatory, wound healing, anti-fungal, antibacterial, anti-tumor and antioxidant properties (Dou et al., 2017; Abu-Taweel and Rudayni, 2022; Singh et al., 2022). The o-methoxyphenol group and the methylenic hydrogen found in Cur are responsible for their antioxidant properties. Cur also plays a role in the bone remodeling process by targeting the NF-κB protein complex, inhibiting the degradation and phosphorylation of NF-κB inhibitor protein (IκB) (Iddir et al., 2022; Kheiridoost et al., 2022). The suppression of the NF-κB protein promotes osteoblast differentiation and inhibits osteoclastic bone resorption (Tong et al., 2016; Yang et al., 2020).

**FIGURE 1:**
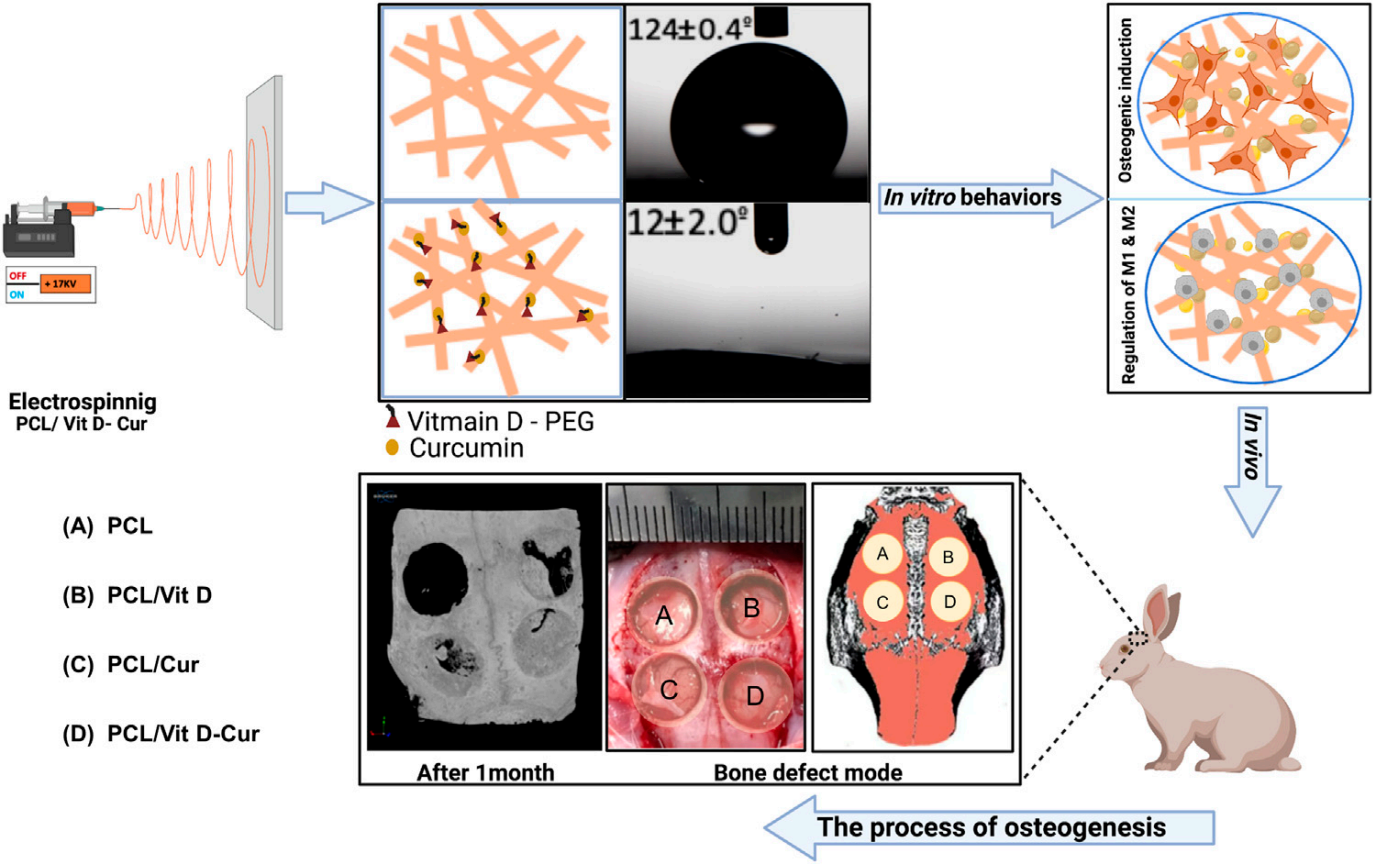
Graph of scaffold fabrication and use for enhancing the anti-inflammatory and bone formation *in vitro* and *in vivo*: **(A)** PCL, **(B)** PCL/Vit D, **(C)** PCL/Cur, and **(D)** PCL/Vit D-Cur.

Additionally, Liang et al. have proved that Cur can reduce the production of CCL3 chemokine in osteoclastic precursors (OCPs), which is responsible for stimulating RANK-independent osteoclastogenesis in bone and bone marrow cells (BMCs) (Lorenzo, 2016; Liang et al., 2020). According to another study, Cur can also decrease the IL-1α induced expression of RANKL in BMSCs, leading to the suppression of osteclastogenesis and inducing apoptosis of osteoclasts (Ozaki et al., 2000; Bharti et al., 2004; Gu et al., 2012; Sahebkar et al., 2016). Moreover, some studies have confirmed the relationship between curcumin and Vit D. The effectiveness of curcumin in improving Vit D deficiency, especially in women with PMS and dysmenorrhea (Arabnezhad et al., 2022). Therefore, in this study, we have utilized the electrospinning method to fabricate a nanofibrous scaffold doped with Vit D and Cur, as shown in Figure 1. This method of slow-release administration will result in fewer side effects and more effective drug delivery. Moreover, the study will investigate the osteogenic effects via dual-delivery of vitamin D and curcumin on post-surgical bone defect.

## 2 Material and method

### 2.1 Preparation of Vit D/Cur loaded electrospinning scaffolds

Briefly, 1 g of PCL (Mn: 70,000 - 90,000) was dissolved in 10 mL of 2,2,2-Trifluoroethanol solution. Meanwhile, Vit D (100 µg and Cur (100 µg) were dissolved in 1 ml of ethanol, respectively. The PCL and Vit D/Cur solution were then fully mixed in a volume ratio of 10:1 for the subsequent electrospinning. Four kinds of PCL scaffolds loaded with/without Vit D, Cur or Vit D/Cur (named as PCL, PCL/Vit D, PCL/Cur, PCL/Vit D-Cur) were fabricated under the conditions of 16.5–17 kV and 1 ml/h flow rate. The collector was covered with a piece of aluminum foil. The distance between the tip of the needle and the collector was about 10 cm. Moreover, the humidity and temperature were maintained at 45%–60% and 25–33°C, respectively.

### 2.2 Fiber characterization

The morphology of different fibrous scaffolds was examined by scanning electron microscopy (SEM, Nova 200 Nano SEM, United States). Samples were dried under nitrogen flow before being coated with gold with a Desk II cold sputter coater for 80 s. At least five areas were randomly selected to test the uniformity of the fibers. All membranes static water contact angle (WCA) was measured using a Contact Angle Analyzer (SDC-200S, China) at ambient temperature. 5.0 μl of water droplets were dropped carefully onto the surface of different membranes. The average WCA value was measured using water droplets at randomly distributed positions. The KBr and scaffold combination was then compacted into sheets at 20 MPa and analyzed using a Fourier transform infrared spectroscopy (FTIR, Thermo scientific, Nicolet 6,700, United States). The reality of crystal phases in the Scaffold composites was estimated using energy-dispersive X-ray diffraction (XRD, SmartLab, Rigaku).

### 2.3 Mechanical properties

The mechanical properties of PCL, PCL/Vit D, PCL/Cur, and PCL/Vit D-Cur scaffolds were investigated using stress-strain curves. The samples were cut into rectangles (2.5 cm × 8 cm) and dried for 24 h in a vacuum at room temperature before being measured. A Testing Machine (4,465, Instron, United States) with a cross-head pulling speed of 5 mm/min was used to measure the dry state. Young’s modulus, tensile strength, and ultimate strain were calculated in static mode.

### 2.4 Release of Vit D and cur *in vitro*

According to previous studies (Alexis et al., 2004; Zhu et al., 2015), a mixture of water and ethanol (80:20) was used as a release medium because Vit D is a fat-soluble vitamin and Cur was slightly soluble in water. Briefly, 50 mg thetheelectrospunn membrane that loaded Vit D and Cur was immersed in 10 ml release medium and then transferred to a shaking incubator (150 rpm) at 37°C. At regular intervals, 1 ml of the release medium was collected and added with the same volume of fresh medium. Afterward, the sample liquids were measured by using a Nanodrop (Thermo Scientific™, Nanodrop 2000 and 2000c, United States) at wavelengths of 240 nm (Vit D) and 427 nm (Cur). A calibration curve was used to determine the accumulative drug release amount, and the data was displayed in a graphical form.

### 2.5 Cell responses of MC3T3-E1 cells

#### 2.5.1 Cell morphology and viability

The samples were placed in a 24-well culture plate for morphology observation and seeded with 8 × 10^3^ cells in each well. After culturing for 2 d, the adherent MC3T3-E1 cells were washed with PBS three times, fixed overnight at 4°C by a 4% fixative solution, and then dehydrated using gradient ethanol (20%, 40%, 60%, 80%, 100%). All samples were sprayed with gold before the final electron microscopic observation. Next, for viability detection, the cells were seeded with 2 × 10^4^ cells in each well (24-well culture plate). After culturing for 7 d, the samples were taken from the incubator. All samples were then incubated with 50 μl CCK-8 solution and 500 μl new medium for another 2 h. Finally, a microplate reader was used to measure the extracts at 450 nm optical density (OD).

#### 2.5.2 Alkaline phosphatase activity

The activity of ALP was determined using commercial ALP and BCA protein detection kits. Briefly, samples were placed in a 24-well culture plate and seeded with 2 × 10^4^ cells in each well. After 7 and 14 d, the adherent cells were via lysed by 1% Triton X-100 solution. Next, 30 μl of cell lysates were extracted for ALP testing. A microplate reader was used to measure the extracts at 520 nm. Additionally, the total protein content of each group was measured using a BCA protein kit at 570 nm.

#### 2.5.3 Mineralization level

The samples were seeded into a 24-well flask with an initial density of 2 × 10^4^ cells/well. After culturing for 14 d, the samples were taken out and washed by PBS. Then, the adherent MC3T3-E1 cells were fixed using 4% formaldehyde for 30 mn and stained with alizarin red dye for another 1 h. Then, all the nanofiber samples were submerged in 10% hexadeceylpyridinium chloride for 40 min. Finally, a microplate reader was used to measure the extracts at OD 540 nm.

### 2.6 Inflammatory/osteogenic gene expression of RAW264.7 cells

The complete RNA was extracted from RAW264.7 cells after 3 d of culture using an RNA extraction kit and then reverse-transcribed to cDNA by a PrimeScriptTM RT reagent kit. The cDNA was amplified in a two-step cycling condition (95°C for 30 s, followed by 39 cycles of 95°C for 5 s and 60°C for 30 s) with a BioRad CFX Manager system. The specific anti-inflammatory (IL-10, IL-1ra) and pro-inflammatory (TNF-α, IL-1β) genes were detected through the BioRad-QuantitativReal-time-PCR system. Furthermore, the mRNA levels of endothelial growth factor (VEGF) and bone morphogenetic protein-2 (BMP-2) were also determined. The primers are shown in Table 1.

**TABLE 1:** The chain reaction for real-time polymerase of some inflammatory genes.

*Target genes*	*Primers*	*Product size (bp)*
IL-10	GCA​TGG​CCC​AGA​AAT​CAA​GG	91
	GAG​AAA​TCG​ATG​ACA​GCG​CC	
IL-1ra	GTG​GCC​TCG​GGA​TGG​AAA​T	116
	CTT​GCA​GGG​TCT​TTT​CCC​AGA	
TNF-α	CAG​GCG​GTG​CCT​ATG​TCT​C	89
	CGA​TCA​CCC​CGA​AGT​TCA​GTA​G	
IL-1β	TGC​CAC​CTT​TTG​ACA​GTG​ATG	138
	TGA​TGT​GCT​GCT​GCG​AGA​TT	
VEGF	GTC​CCA​TGA​AGT​GAT​CAA​GTT​C	209
	TCT​GCA​TGG​TGA​TGT​TGC​TCT​CTG	
BMP-2	GGG​ACC​CGC​TGT​CTT​CTA​GT	154
	TCA​ACT​CAA​ATT​CGC​TGA​GGA​C	
GADPH	F: 5′- CTC​GTC​CCG​TAG​ACA​AAA​TGG​T -3	131
	R: 5′- GAG​GTC​AAT​GAA​GGG​GTC​GTT -3′	

### 2.7 Cellular responses of osteoblasts in macrophage-mediated conditioned medium

RAW264.7 cells were first cultured on different samples for 48 h. The supernatants (conditioned medium) were centrifuged at 1,000 rpm for 5 min at 4°C and filtered by a 0.2-micron-pore-size filter to eliminate cell debris. After that, the MC3T3-E1 cells were seeded in a 48-well flask and cultured with the above macrophage-mediated conditioned medium. The cell viability (7 d), ALP activity (4 & 7 d), and mineralization (14 d) of MC3T3-E1 cells were then measured with reference to section 2.5.1–2.5.3. In addition, the expression of some osteogenic genes [runt-related transcription factor 2 (Runx2), ALP, osteopontin (OPN), and collagen I (COL-I)] in MC3T3-E1 cells were ultimately evaluated at 7 d according to section 2.6. The primers are shown in Table 2.

**TABLE 2:** The chain reaction for real-time polymerase of some osteogenic genes.

*Target genes*	*Primers*	*Product size (bp)*
Runx2	F: 5′-GCC​GTA​GAG​AGC​AGG​GAA​GAC-3′	150
	R: 5′-CTG​GCT​TGG​ATT​AGG​GAG​TCA​C-3′	
ALP	F: 5′-AGC​GAC​ACG​GAC​AAG​AAG​C-3′	183
	R: 5′-GGC​AAA​GAC​CGC​CAC​ATC-3′	
OPN	F: 5′-GAC​AGC​AAC​GGG​AAG​ACC-3′	216
	R: 5′-CAG​GCT​GGC​TTT​GGA​ACT-3′	
Col I	F: 5′-CCT​GAG​CCA​GCA​GAT​TGA-3′	106
	R: 5′-TCC​GCT​CTT​CCA​GTC​AG-3′	
GADPH	F: 5′- CTC​GTC​CCG​TAG​ACA​AAA​TGG​T -3	131
	R: 5′- GAG​GTC​AAT​GAA​GGG​GTC​GTT -3′	

### 2.8 *In vivo* evaluation

#### 2.8.1 Surgical procedures and implantation

We employed twenty White Rabbits (New Zealand) with an average weight of between (2.5–3.5 kg) for this experiment. The experiment was carried out according to the regulations of the Animal Ethics Committee of Wenzhou Medical University. Before the skin incision, 70% ethanol and povidone-iodine were used to disinfect the skin. After anesthesia and regular preparation, 8 mm bone defects were made on the skull and covered with different electrospun membranes. Following the surgical procedure, post-surgical preventive antibiotics (gentamicin/penicillin) were given subcutaneously for 5 d to minimize the risk of infection.

#### 2.8.2 Micro-CT analysis

After 4 weeks of implantation, the head of the rabbit was taken and fixed for a week in 10% formalin. A Micro-CT scanner (Skyscan 1,176, Wenzhou Medicine University, China) was used to examine the newly produced bone at the defect sites. 3D models were made by utilizing equipped software which was utilized to assess the bone tissue density (BV/TV), connection density (Conn.D), trabecular thickness (Tb.Th) and trabecular separation (Tb.Sp).

### 2.9 Statistical analysis

The program (Graphpad Prism v8.2.1) was used for data analysis. A one-way ANOVA followed by Tukey multiple comparisons was used to compare the groups in this study. The statistical significance was defined as a *p < 0.05 and **p < 0.01.

## 3 Results

### 3.1 Fibers characterizations

**FIGURE 2:**
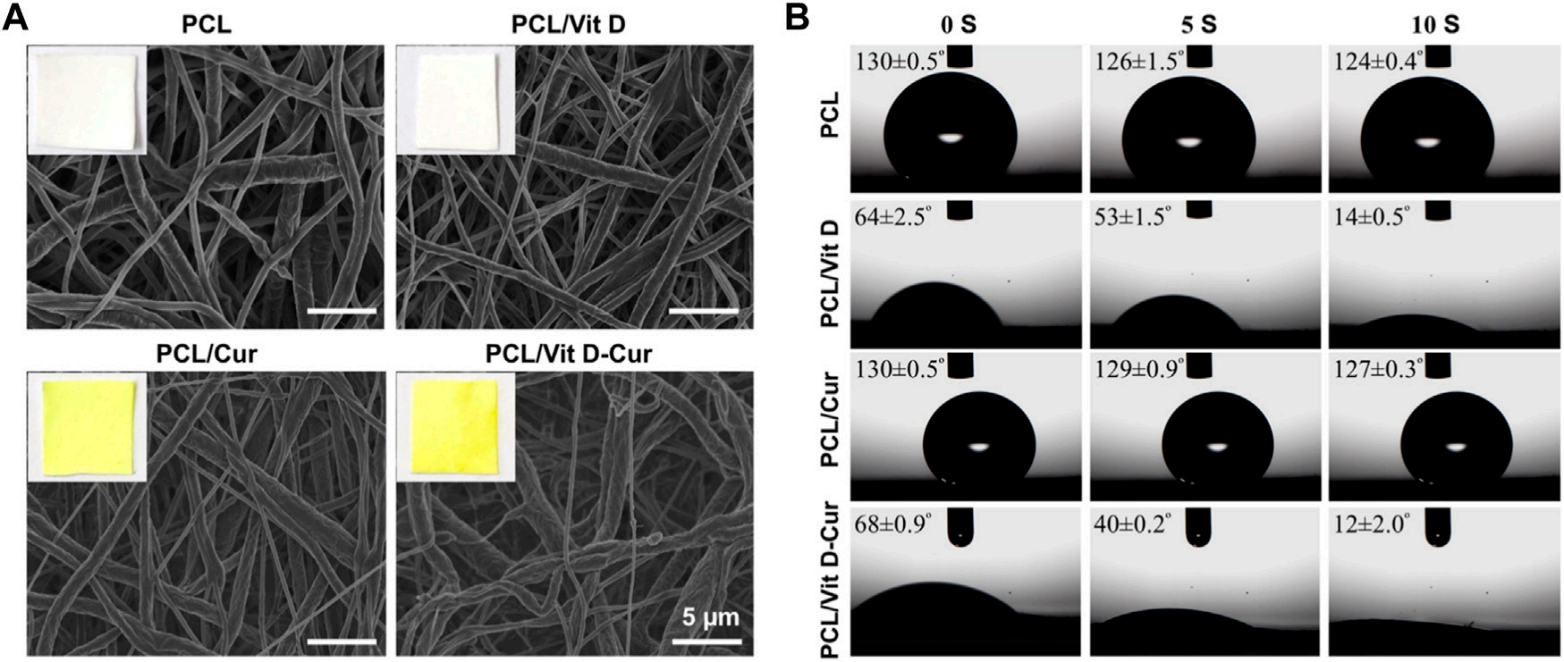
**(A)** SEM images, and **(B)** water contact angle of different scaffolds.

**FIGURE 3:**
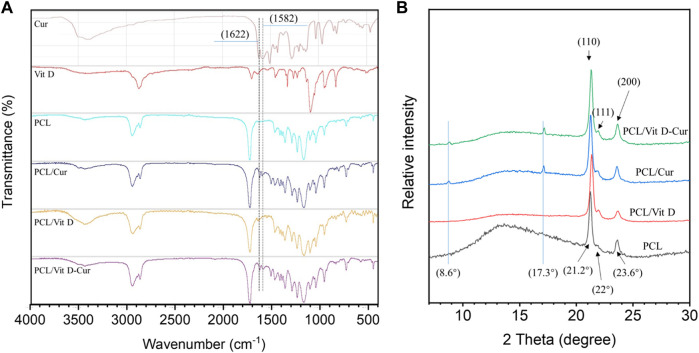
**(A)** FTIR spectra and **(B)** XRD patterns of different scaffolds.

SEM examinations were conducted to investigate the fiber morphology. As shown in Figure 2A, there was no significant change in the morphology of fibers produced from PCL, PCL/Vit D, PCL/Cur and PCL/Vit D-Cur scaffolds. All scaffolds were properly linked fibers. The PCL and PCL/Cur electrospinning nanofiber scaffolds were hydrophobic (Figure 2B). Within 10 s, the contact angle of PCL was 124.4°, and of PCL/Cur was 127.3°. In addition, Vit D was considered hydrophobic, but PEG modification significantly enhanced its hydrophilicity, resulting in a water contact angle of 14.1° for PCL/Vit D membrane at 10 s. Additionally, when Vit D and Cur were co-doped with PCL fibers, the corresponding PCL/Vit D-Cur samples also showed good hydrophilicity (about 12.2°), which is very important for bone repair materials. The FTIR data of Figure 3A were measured using KBr pellet method, and the spectra of Cur are downloaded from the Chemistry Database (Shanghai Institute of Organic Chemistry of CAS., http://www.organchem.csdb.cn). As shown in Figure 3A, the PCL, PCL/Vit D, PCL/Cur, and PCL/Vit D-Cur scaffolds exhibit similar adsorption features. The peak at 2,940 and 2,860 cm^−1^ is assigned to the asymmetric and symmetric stretching vibration of CH_2_, and the peaks at 1723 cm^−1^ are assigned to the C=O stretching vibration. The peaks at 1,622 and 1,583 cm^−1^ can be attributed to the C=C stretching vibration of the aromatic ring, indicating the successful loading of Cur into the PCL scaffold. However, since the chemical structure of PEG-Vit D is consistent with that of PCL, their FTIR spectra are almost identical. XRD was used to detect the crystalline structures of various scaffolds. As shown in Figure 3B, there is no obvious trend in the overall crystallinity of PCL nanofibers loaded with Vit-D and Cur molecules. However, compared to PCL, the decrease of the peak at 12–17° and the increase of the (110) peak (2θ = 21.2°) and (110) peak (2θ = 22°) of PCL/Vit-D, PCL/Cur and PCL/Vit D-Cur scaffold suggested an increase in crystalline perfection. This indicated that Cur and Vit D were successfully incorporated into the PCL scaffold and had a weak effect on the crystallization of the PCL scaffold. In addition, the characteristic peak appeared at around 8.6 and 17.3°, confirming the presence of Cur in the PCL/Cur and PCL/Vit D-Cur groups (Ching et al., 2019).

**FIGURE 4:**
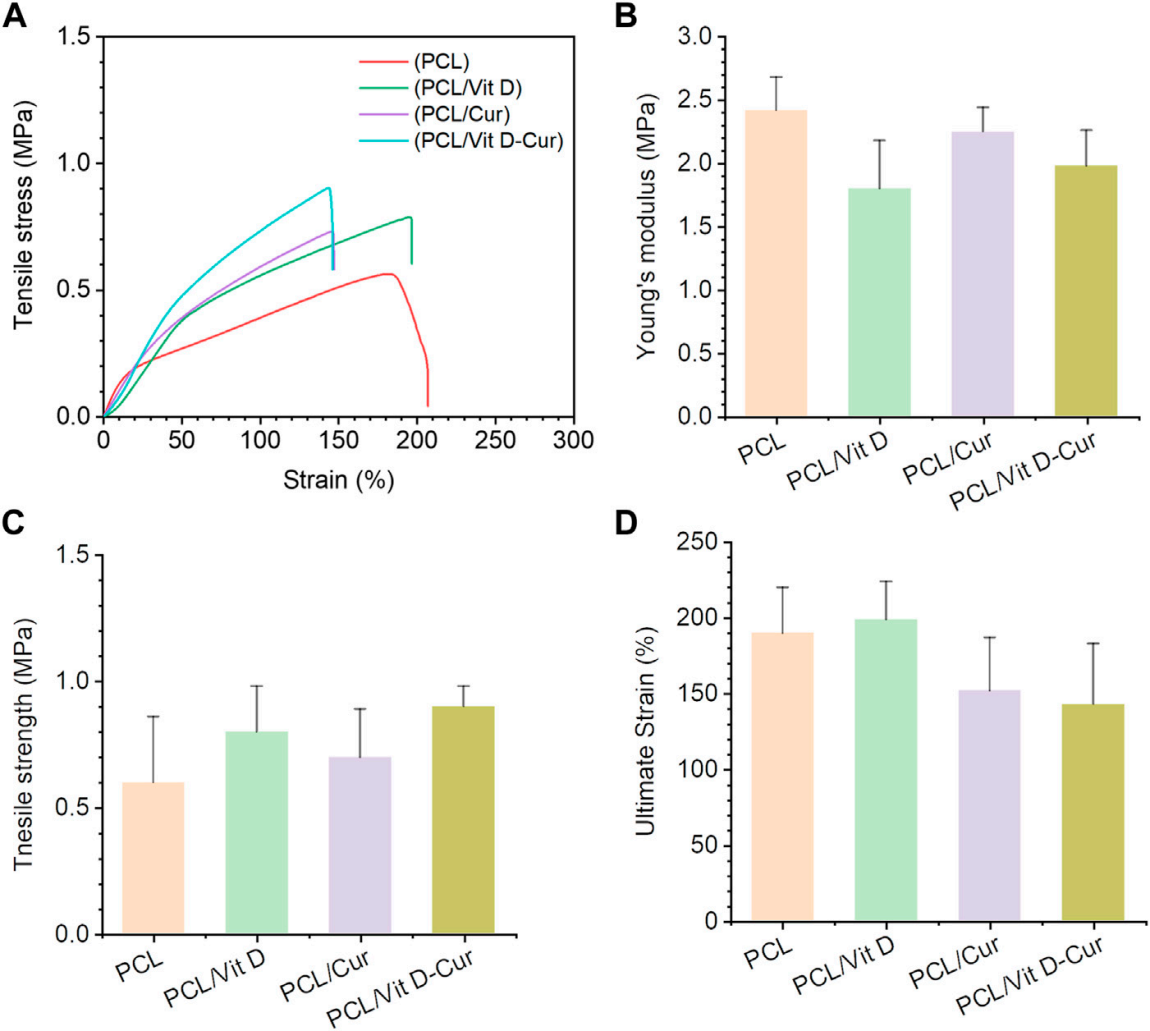
Mechanical characterization of PCL, PCL/Vit D, PCL/Cur, PCL/Vit D-Cur scaffolds **(A)** representative stress-strain curves of scaffolds recorded from a tensile test; **(B)** Young’s modulus, **(C)** tensile strength, and **(D)** ultimate strain calculated from the stress-strain curves. Error bars represent mean ± SD for *n* = 3.

To investigate the mechanical characteristics of PCL, PCL/Vit D, PCL/Cur, and PCL/Vit D-Cur scaffolds, stress-strain measurements were performed. The stress-strain curve Figure 4A showed a direct relationship between the mechanical performance of different scaffolds. The results showed that the tensile strength of the PCL/Vit D-Cur group was slightly increased, but the ultimate strain was slightly decreased compared to the other groups (Figures 4C,D). The changes in mechanical properties may be due to the interconnection and entanglement of the PCL scaffold with Vit D and Cur, as well as the effect of Vit D and Cur on the crystallization of the PCL scaffold.

### 3.2 Release profiles

**FIGURE 5:**
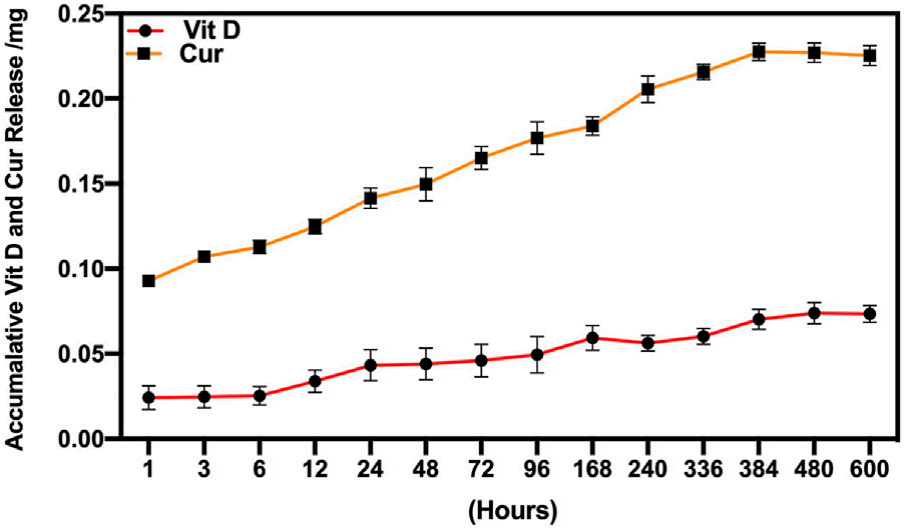
Vit D and Cur drug release profiles from PCL/Vit D-Cur samples within 25 d.

In this study, both Vit D and Cur showed an overall higher release in the mixture medium. The resulting release curves are shown in Figure 5. The release of Vit D and Cur in PCL followed a constant sustained pattern, although Cur showed a higher release amount compared to Vit D release. The drug release profile confirmed that the Vit D and Cur released from the scaffolds served up to 25 days.

### 3.3 MC3T3-E1 cell behaviors on different nanofiber membranes

**FIGURE 6:**
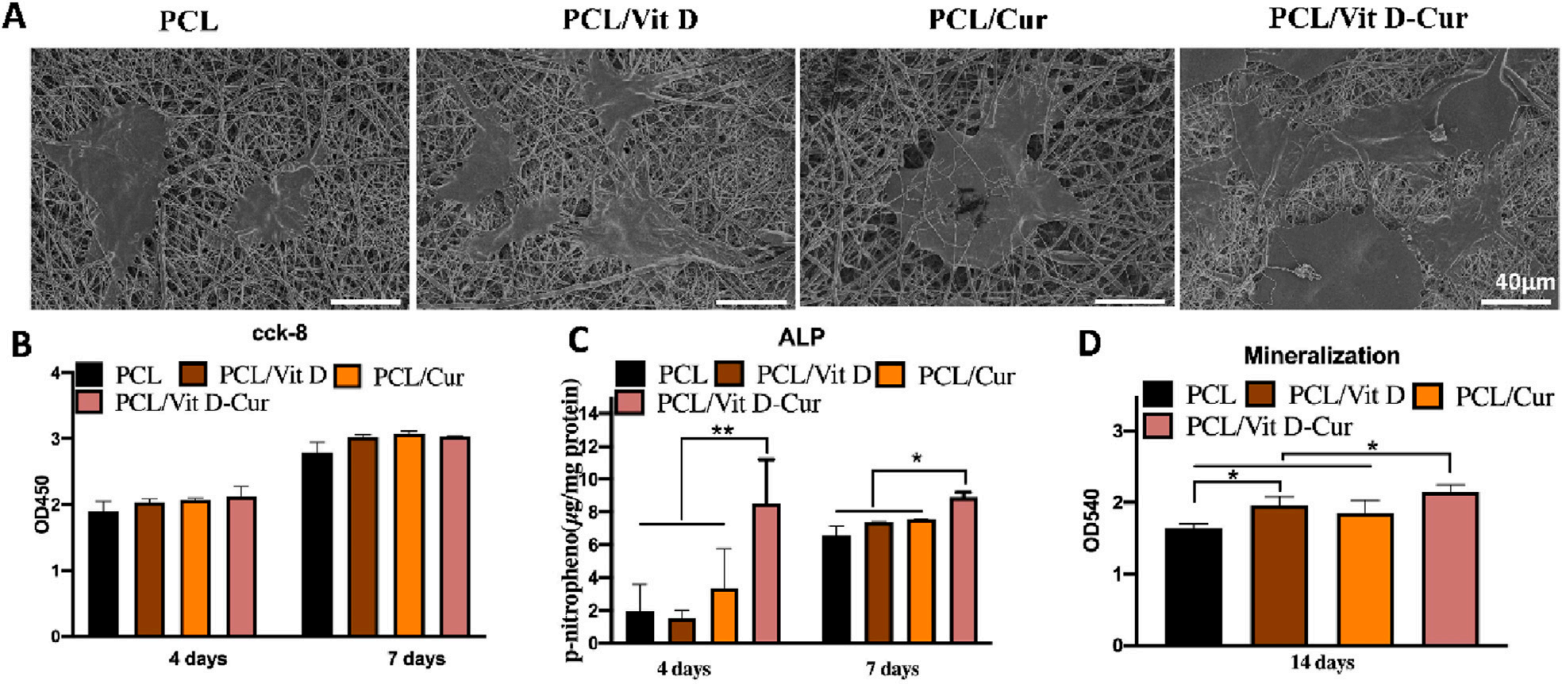
**(A)** The morphology of MC3T3-E1 cells on different scaffolds determined by SEM; **(B)** viability of MC3T3-E1 cells after 4 and 7 d; **(C)** the quantitative ALP analysis after 4 and 7 d; and **(D)** the quantitative mineralization results after 14 d (*p < 0.05, **p < 0.01).

The SEM results showed that cells in the PCL/Vit D-Cur group were more widely spread compared to other groups, which means that both Vit D and Cur could promote cell spreading and improve biocompatibility (Figure 6A). The assay of CCK-8 was performed to evaluate cell viability after 4, and 7 d of culturing and the results are illustrated in Figure 6B. The results demonstrate no cytotoxicity and no significant differences in cell viability. We also examined the ALP activity and the formation of the calcium matrix. Firstly, ALP was utilized to estimate the efficacy of PCL/Vit D-Cur to influence initial osteogenic differentiation. The normalized activity of ALP toward all samples was increased from to 7 d, as shown in Figure 6C. On days 4 and 7, the ALP activity in PCL/Vit D-Cur group was significantly higher than that of all other groups. On day 7, PCL/Vit D and PCL/Cur groups were significantly higher than the PCL group. Furthermore, according to the quantitative ARS findings, the extracellular matrix mineralization of the PCL/Vit D-Cur scaffold group was better than in the other three groups (Figure 6D).

### 3.4 Inflammatory responses of macrophages

**FIGURE 7:**
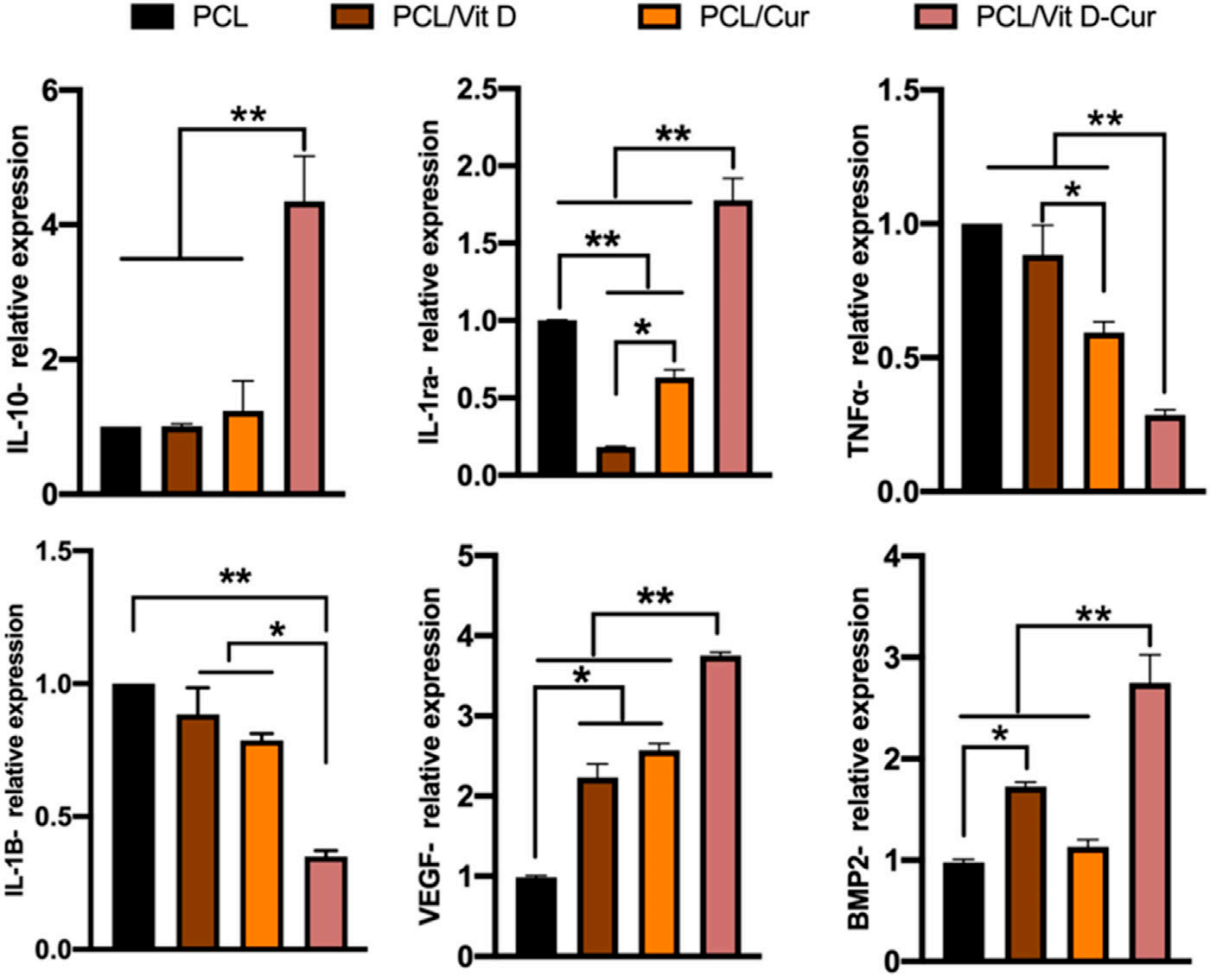
The expression of anti-inflammatory (IL-10, IL-1ra), pro-inflammatory (TNF-α, IL-1β), and osteogenic (BMP-2, VEGF) genes by RAW264.7 cells grown on different scaffolds. The values were normalized by GAPDH (*p < 0.05, **p < 0.01).

Figure 7 shows the expression of some inflammatory genes, including anti-inflammatory genes IL-10/IL-1ra and pro-inflammatory gene TNF-α/IL-1β. The expression of four genes in the PCL/Vit D-Cur group and other groups was significantly different. PCL/Vit D-Cur substrates significantly increased IL-10 and IL-1ra expression while decreased IL-1β and TNF-α expression. Besides, macrophages cultured on PCL/Vit D-Cur enhanced the expression of angiogenic and osteogenic genes (VEGF and BMP-2), as shown in Figure 7.

### 3.5 Effects of RAW cells condition medium on osteogenesis behaviors of MC3T3

**FIGURE 8:**
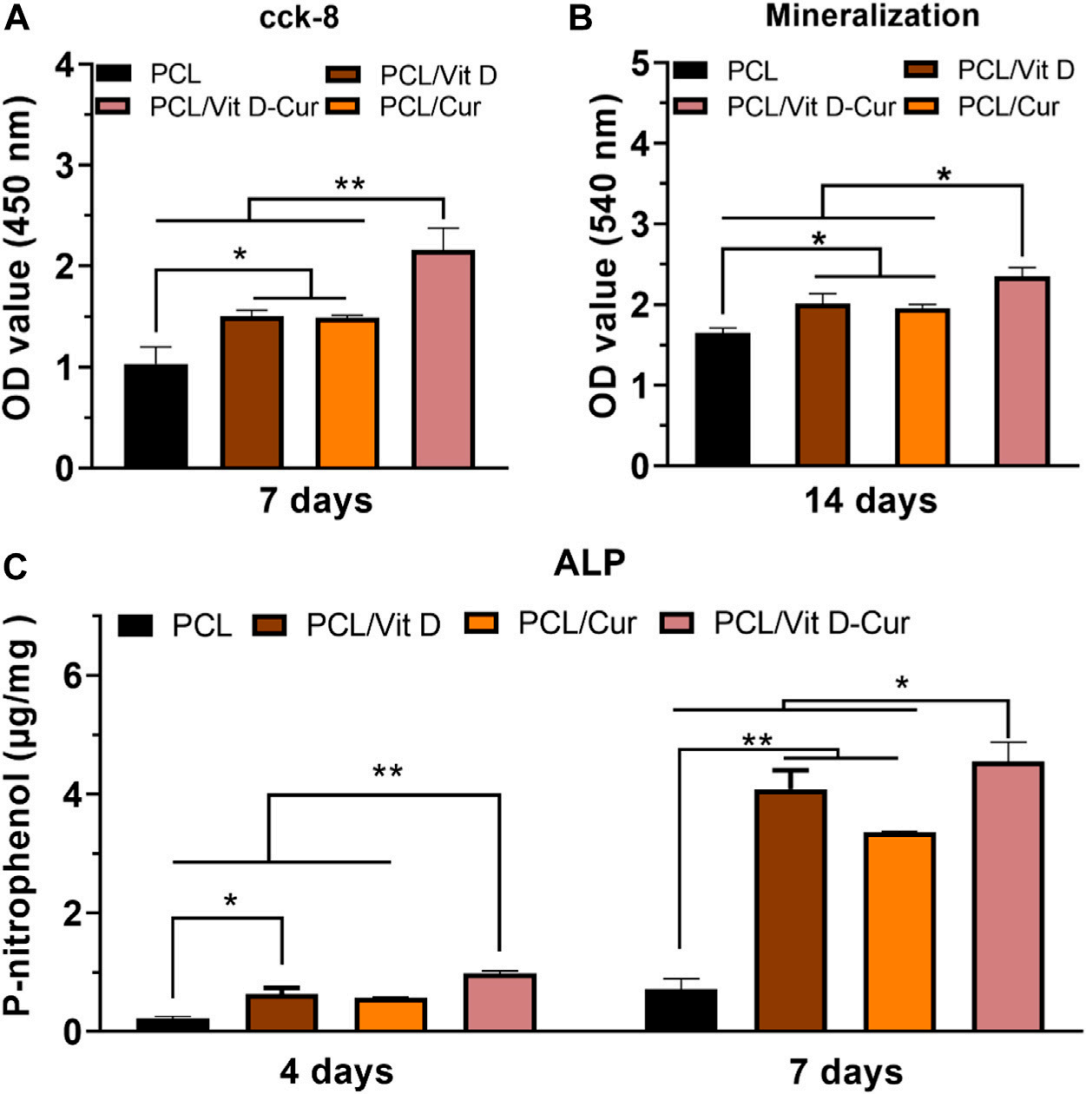
**(A)** The viability, **(B)** ALP activity and **(C)** mineralization of MC3T3-E1 cells cultured in condition medium (CM) after 4, 7 or 14 d (*p < 0.05, **p < 0.01).

**FIGURE 9:**
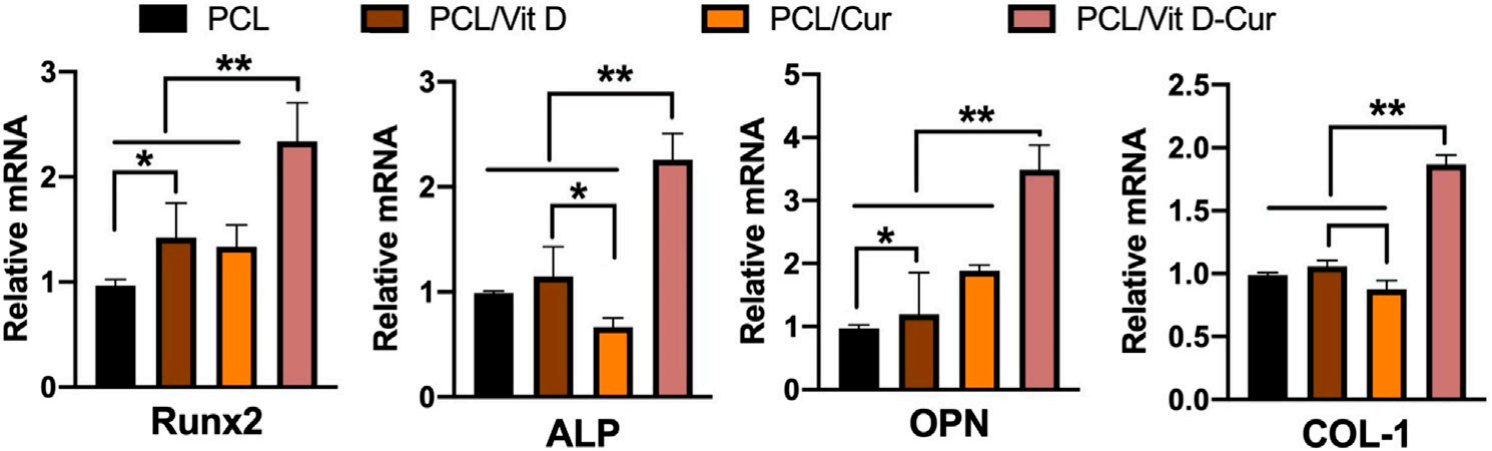
The relative expression of Runx 2, ALP, OPN, and COL I genes was analyzed by RT-PCR. The values were normalized by GAPDH (*p < 0.05, **p < 0.01).

In this study, the conditioned medium of RAW264.7 cells seeded on different membranes was collected and used to activate MC3T3-E1 cells. As expected, PCL/Vit D-Cur exhibited the greatest effect on cell viability, ALP activity, and mineralization level (Figure 8). There was a significant difference between PCL/Vit D-Cur and the other three groups. Moreover, the mRNA levels of Runx2, ALP, OPN, and COL-І in the PCL/Vit D-Cur group were significantly higher than those in the other groups after culturing for 7 d in the conditioned medium (Figure 9).

### 3.6 Micro-CT analysis

**FIGURE 10:**
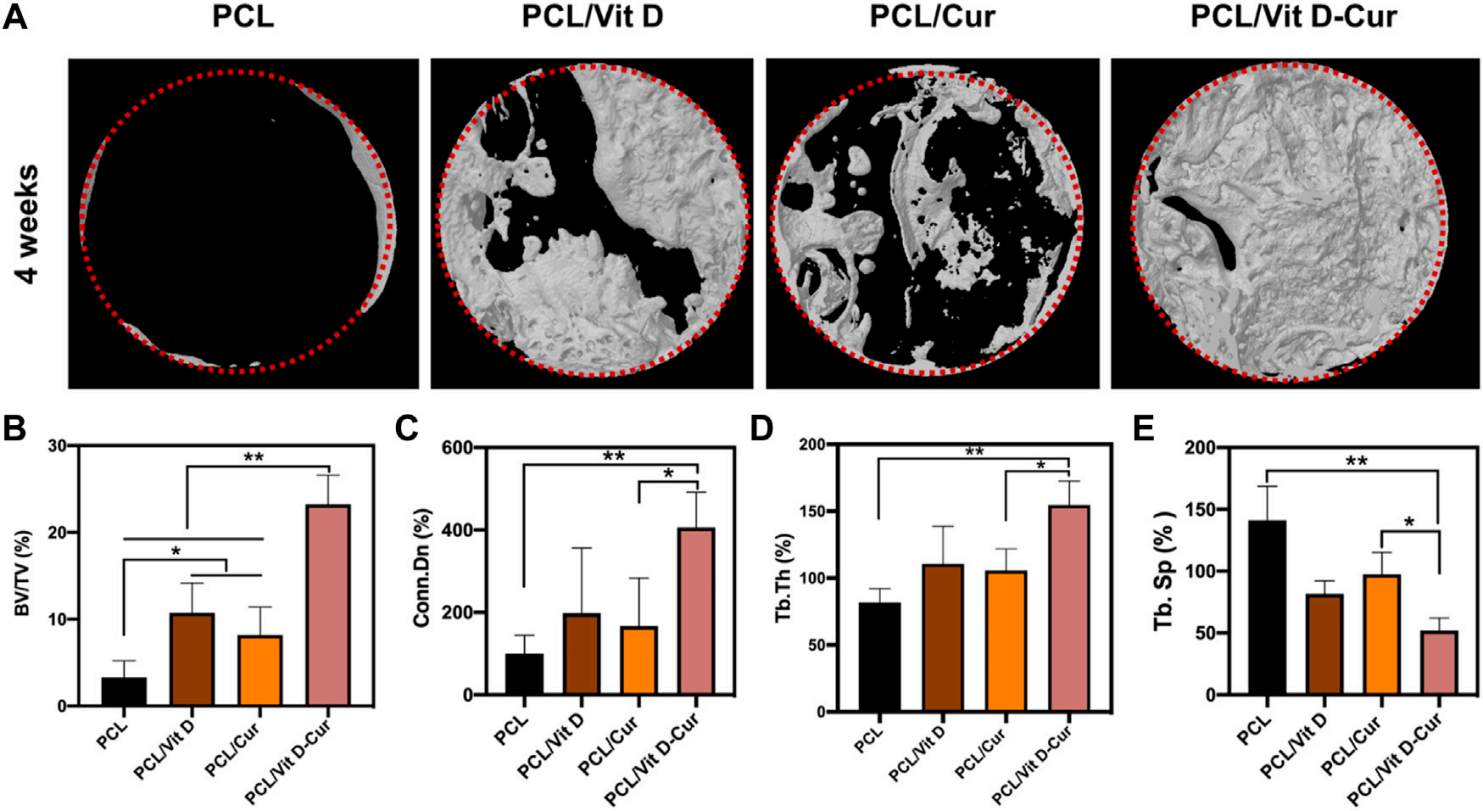
**(A)** The Micro-CT 3D images after 1 month of scaffold implantation and the statistical analysis of BV/TV **(B)**, Conn. Dn **(C)**, Tb.Th **(D)**, and Tb. Sp **(E)** (*p < 0.05, **p < 0.01).

The micro-CT orthography was used to portray the three-dimensional network of the newly formed bones. The findings revealed that dual Vit D and Cur releases improved bone formation after 4 weeks of operation. The scaffold loaded with Vit D and Cur demonstrated the most compatible osseointegration within 4 weeks compared to PCL, PCL/Vit D, and PCL/Cur scaffolds. The bone in the defect area of the PCL/Vit D-Cur scaffold shows a significantly higher ratio of bone tissue formation, as shown in Figure 10A. Furthermore, as shown in Figures 10B–D, the BV/TV, Conn. D and Tb. The PCL/Vit D-Cur group was significantly higher than the other groups (*p* < 0.05 or/and <0.01). Additionally, the Tb. Sp around the PCL/Vit D-Cur scaffolds was significantly less than other scaffolds, as shown in Figure 10E.

## 4 Discussion

The present research proposed a novel dual-drug scaffold for osteogenic and anti-inflammatory co-delivery to guide bone regeneration in bone deformities. The rapid advancements in material science and biotechnology through accessible support materials make the bone tissue scaffolds continuously evolve to enhance cell adhesion and proliferation, promoting bone regeneration by continuously releasing bio-molecules (Alonzo et al., 2020; Wu et al., 2020). The scaffolds have been used as a drug carrier to control therapeutic delivery and enhance the proliferation of osteoblasts (Sarigol-Calamak and Hascicek, 2018). Moreover, the binding of Vit D to the PEG and combining with Cur on one scaffold enhanced its hydrophilicity. The hydrophilicity of the scaffold plays an important role in biological effects, which could enhance the appropriate for blood coagulation, osteogenic induction, and osteointegration (He et al., 2021a; He et al., 2021b). Therefore, mimicking the *in vivo* environment and the natural physiological microenvironment, drug release testing at pH 7.4 was required to ensure adequate drug delivery in the defect and complete osseointegration success (Nourbakhsh et al., 2020). Weak alkaline conditions benefit from polyphenols’ ionization and inhibit the release of Cur. PEG-Vit D makes the PCL scaffold from hydrophobic to hydrophilic so that Cur can be released rapidly in the early stage of implantation to exert biological effects (Rahmati et al., 2019). In this research, the *in vitro* release kinetics of Vit D and Cur confirmed that both had a controlled and prolonged drug delivery from the scaffold at pH 7.4 for 25 d. Also, some studies have shown that the drug’s molecular weight plays an important role in the drug release amounts (Mittal et al., 2007; García-Manrique et al., 2020; Ahmadi et al., 2022). Here, the release amount of Vit D was lower than that of Cur, where the molecular weight of PEG-Vit D was 5,000 Da while Cur was 368.38 Da.

Vit D is an essential vitamin for bone mineral density that offers excellent prospects in the prevention and treatment of osteoporosis and overall maintenance of bone health (Lehouck et al., 2011; Aggarwal and Bains, 2020). Recently, several investigations have confirmed the importance of Vit D action in the osseointegration process, and they suggest that the deficiency of Vit D negatively affects bone regeneration, and the osseointegration of implants might be impaired (Dvorak et al., 2012; Liu et al., 2014). It is proved that an inadequate serum level of Vit D is also closely associated with bone mineral density (Kelly et al., 2009). During the osseointegration period, Vit D can affect the result through its effect on the immune system. According to epidemiological studies, calcitriol can affect both innate and acquired immunity by promoting macrophage function, enhancing chemotaxis and phagocytosis, and producing cytokines and other immunomodulatory peptides (Insua et al., 2017). The association of Vit D deficiency with reduced antimicrobial activity of cells and increased risk of bacterial infections is reported by many studies. They indicate the importance of Vit D adequacy at the clinical level during osteointegration, and later graft union stages (Javed et al., 2016).

On the other hand, the property of Cur, which has been widely studied, has no adverse effects on the human body, even when taken in large doses of 2–12 g/day (Gupta et al., 2013). Furthermore, Cur has been shown to have a significant role in osteoblast differentiation as well as in the prevention of RANKL-induced osteoclast resorption, which may explain its outstanding performance in restoring bone health (Ji et al., 2013; Banez et al., 2020). Besides its anti-inflammatory properties, Cur may also have anti-inflammatory benefits via inhibiting the formation of reactive oxygen species (ROS) (Hardwick et al., 2021). Therefore, scaffolds loaded with Vit D and Cur represent a promising and safer alternative for treating bone defects by providing a higher local drug concentration and relieving adverse side effects or cytotoxicity to healthy cells. Our findings showed that PCL/Vit D-Cu scaffold does not exhibit any toxicity toward osteoblast and promotes its viability, proliferation, ALP activity, mineralization, and osteogenic genes expression (Runx 2, ALP, OPN, COL-1). In addition, the PCL/Vit D-Cu scaffold contributes effectively to increasing and regulating M2-related genes such as (IL-10) and (IL-1ra) processes by performing an anti-inflammatory role and producing anti-inflammatory cytokines.

Moreover, it decreases M1-related genes such as (TNF-α) and (IL-1β) respond to inflammatory signaling by performing a pro-inflammatory role, which may improve the anti-inflammatory effects and promote bone tissue regeneration. These findings verify the rapid augmentation of anti-inflammatory and osteogenesis by enhancing the bone mineral density as well as increasing the mature bone microstructure through the Vit D and Cu. In conclusion, the PCL/Vit D-Cu membrane could be a promising drug candidate for the therapy for bone tissue regeneration, which may overcome the limitation faced by current bone tissue regeneration.

## 5 Conclusion

This study aimed to utilize dual drugs-loaded nanofiber membranes with the advantage of local delivery of bioactive drugs to accelerate osteogenesis. XRD and FTIR showed that Vit D and Cur were successfully incorporated into PCL nanofibers. Although Vit D and Cur had some effect on the mechanical properties of PCL, the target membrane was shown to have sufficient mechanical stability for bone repair applications. In addition, PEG-Vit D significantly improved the hydrophilicity of PCL nanofiber membranes. The surface was becoming hydrophilic and improved the biocompatibility of the PCL scaffolds and the release of Cur.

Furthermore, in vitro-related assays confirmed that the nanofibers with a prolonged release profile of Vit D/Cur had good cytocompatibility and improved cell proliferation/differentiation. In addition, the target membrane polarized more RAW264.7 cells toward alternatively activated macrophages (M2), as shown by upregulated M2 expression markers (IL-10, IL-1ra) and downregulated M1 expression markers (TNF-α, IL-1β) in the cells. Also, the PCL/Vit D-Cur significantly increases the VEGF and BMP-2 expression compared to other groups.

Moreover, MC3T3-E1 cells cultured in a conditioned medium from the RAW264.7 cells showed significantly promoted ALP, Red stain, and osteogenic-related genes. Furthermore, the *in vivo* results demonstrated that after 1 month of membrane implantation in a rabbit model, the osteoinductive properties of PCL scaffold loaded Vit D/Cur significantly improved bone reformation. Hence, this study offers a simple yet successful development of scaffolds for bone regeneration.

## Data Availability

The original contributions presented in the study are included in the article/supplementary material, further inquiries can be directed to the corresponding authors.
