# Correction: Vitamin D and curcumin-loaded PCL nanofibrous for engineering osteogenesis and immunomodulatory scaffold

**DOI:** 10.3389/fbioe.2025.1664137

**Published:** 2025-09-16

**Authors:** Abdullrahman M. Al-Bishari, Bilal A. Al-Shaaobi, Aisha A. Al-Bishari, Mohammed A. Al-Baadani, Liang Yu, Jiating Shen, Lei Cai, Yiding Shen, Zhennan Deng, Peng Gao

**Affiliations:** ^1^ School and Hospital of Stomatology, Wenzhou Medical University, Wenzhou, China; ^2^ College of Dentistry, University of Science and Technology, Sanaa, Yemen; ^3^ School Hospital of Stomatology, Zhejiang Chinese Medical University, Hangzhou, China

**Keywords:** electrospinning, vitamin D, curcumin, anti-inflammatory, bone regeneration

In the published article, there was an error in Figure 6A as published. We mistakenly used the image from the PCL group for the PCL/Cur group. The corrected Figure 6A and its caption appear below.

**FIGURE 6:**
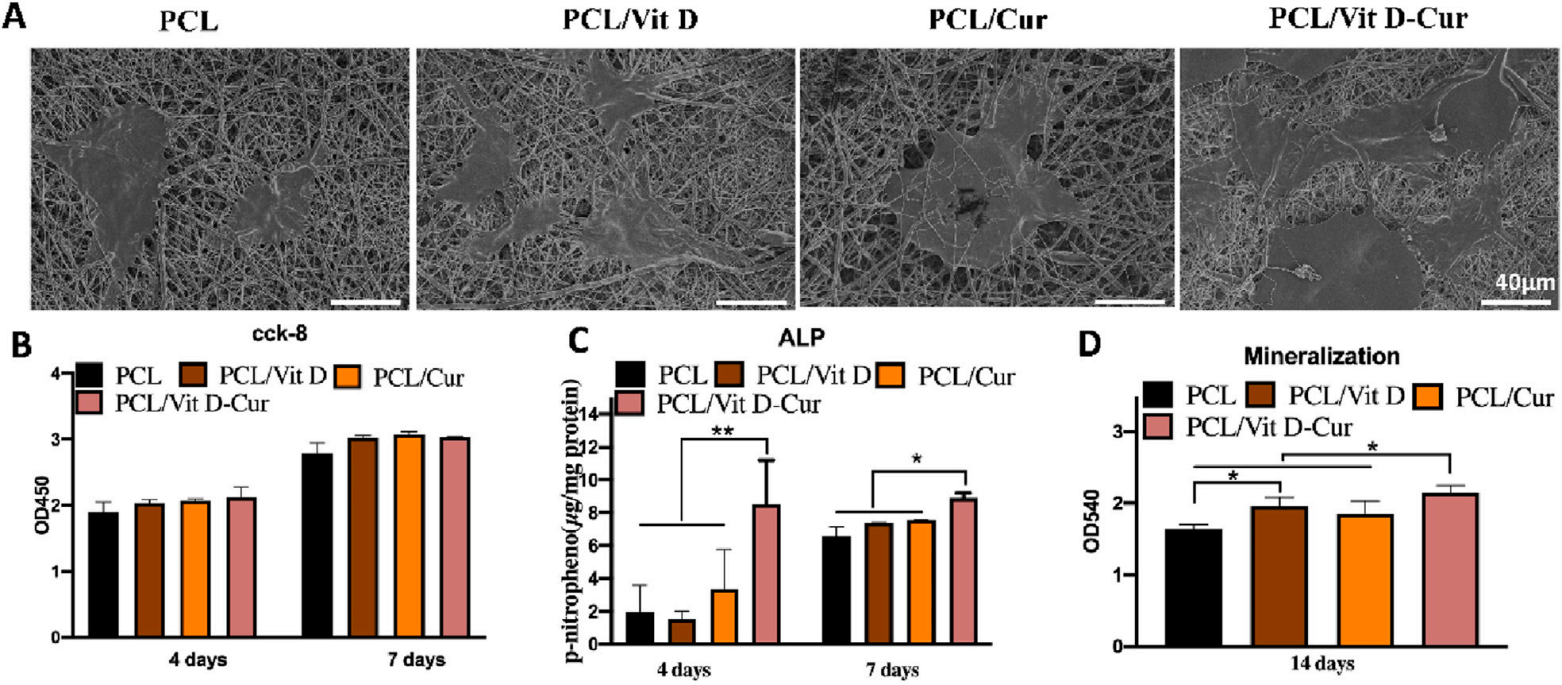
**(A)** The morphology of MC3T3-E1 cells on different scaffolds determined by SEM; **(B)** viability of MC3T3-E1 cells after 4 and 7 days; **(C)** the quantitative ALP analysis after 4 and 7 days; and **(D)** the quantitative mineralization results after 14 days (*p < 0.05, **p < 0.01).

The original article has been updated

